# Leukoencephalomyelopathy in cats linked to abnormal fatty acid composition of the white matter of the spinal cord and of irradiated dry cat food

**DOI:** 10.1111/jpn.13139

**Published:** 2019-06-07

**Authors:** Ted S. G. A. M. van den Ingh, Guy C. M. Grinwis, Ronald Jan Corbee

**Affiliations:** ^1^ TCCI Consultancy Utrecht The Netherlands; ^2^ Department of Pathobiology, Faculty of Veterinary Medicine Utrecht University Utrecht The Netherlands; ^3^ Department of Clinical Studies in Companion Animals – Clinical Nutrition, Faculty of Veterinary Medicine Utrecht University Utrecht The Netherlands

**Keywords:** cat, fatty acid composition, irradiated food, leukoencephalomyelopathy, white matter

## Abstract

Four outbreaks of leukoencephalomyelopathy in colonies of SPF cats on a long‐term diet of irradiated dry cat food were observed in the Netherlands between 1989 and 2001. As a primary defect in myelin formation was suspected to be the cause of the disease and myelin consists mainly of lipids and their fatty acids, we investigated the fatty acid composition of the white matter of the spinal cord of affected and control cats and of irradiated and non‐irradiated food. The irradiated food had low levels of alpha‐linolenic acid compared to linoleic acid as well as a high total omega‐6:omega‐3 ratio of 7:1 in the irradiated and of 2:1 in the non‐irradiated food. The white matter of the spinal cord showed low levels of linoleic acid and absence of alpha‐linolenic acid in affected cats as well as absence of lignoceric and nervonic acid in both affected and control cats. These abnormalities in fatty acid composition of the white matter of the spinal cord may reflect an increased need for alpha‐linolenic acid as a substrate for longer chain omega‐3 fatty acids to compose myelin and thus indicate a particular species sensitivity to dietary deficiency in omega‐3 polyunsaturated fatty acids, particularly alpha‐linolenic acid in cats. Our findings indicate that abnormalities in fatty acid metabolism in myelin play an essential role in the pathogenesis of this acquired form of leukoencephalomyelopathy in cats.

## 1 INTRODUCTION

Leukoencephalomyelopathy has first been described in young cats suffering from a progressive form of ataxia between 1969 and 1980. These cats originated from specific pathogen‐free (SPF) colonies and the neurological condition affected up to 40% of litters in a colony (Palmer & Cavanagh, 1995). Although initially an infectious or toxic cause was suggested, extensive investigations for such aetiologies were consistently negative. In a series of outbreaks of a clinically and pathologically similar syndrome between 1998 and 2001, reported from Ireland, 25%–40% of cats in SPF colonies were affected (Cassidy et al., 2007). The authors provided initial circumstantial evidence that the condition was associated with the exclusive feeding of the cats of a gamma‐irradiated dry cat food. Their hypothesis was further substantiated as after supplementation and replacement of the irradiated food with pasteurized proprietary tinned cat food no further cases occurred, and definitely proven by the experimental induction of leukoencephalomyelopathy in cats by long‐term (140–174 days) feeding of a gamma‐irradiated dry cat food (Caulfield et al., 2009). Similar cases of leukoencephalomyelopathy, both clinically and pathomorphologically, have been observed in Australia between 2008 and 2009 in 87 private owned cats eating imported, irradiated dry cat food (Child, Foster, Fougere, Milan, & Rozmanic, 2009). From the studies of Duncan, Brower, Kondo, Curlee, and Schultz (2009) on demyelinization and remyelinization of the central nervous system in cats on a long‐term diet of irradiated dry cat food, it is evident that the myelin sheath is primarily affected and axonal damage is initially absent.

Although the irradiated dry cat foods have decreased vitamin A levels and increased peroxide levels, and increased peroxide levels were found in the liver and spinal cords of the affected cats (Caulfield et al., 2009), a definite cause/effect relationship could not be established.

Depletion of vitamin A after gamma‐irradiation has been suggested as a possible contributing factor of leukoencephalomyelopathy in cats (Cassidy et al., 2007; Caulfield et al., 2009) However, the reported (6,230–6,900 IU/kg dry matter basis) vitamin A concentration in the irradiated food in these cases was low, but within normal reference range for the adult cat according to FEDIAF nutritional guidelines publication December 2018 (3,330–400,000 IU/kg dry matter basis). Moreover, cats with leukoencephalomyelopathy associated with irradiated food fail to develop clinical signs or lesions that are typical for vitamin A deficiency, such as obvious squamous metaplasia of the epithelia of conjunctiva, trachea, salivary ducts and uterus (Gershoff, Andrus, Hegsted, & Lentini, 1957).

In a more recent study, the peroxide levels in commercial dry animal diets were increased 14‐ to 25‐fold after high single‐dose gamma‐irradiation of 38.4–48.7 kGy, and cats with leukoencephalomyelopathy had increased peroxide levels in liver and spinal cords (Caulfield, Cassidy, & Kelly, 2008). Hydrogen peroxide is toxic to many cells if it comes into contact with reduced forms of certain metal ions, forming the highly reactive hydroxyl radical that acts to cause lipid peroxide formation (Halliwell & Gutteridge, 1985). However, the mechanism by which changes induced in foods then result in damage to the white matter of the spinal cord and brain in cats is not clear (Child et al., 2009). Moreover, irradiated dry dog and rodent diets which have similar levels of peroxides never caused nervous signs or lesions in these animals (Caulfield et al., 2008).

The present paper describes the pathomorphological findings in four outbreaks of leukoencephalomyelopathy in colonies of SPF cats fed irradiated dry cat food in the Netherlands between 1989 and 2001. Because a definite cause for the disease has not been established until now, and the demyelinating lesions and their distribution throughout the white matter of the central nervous system in these cats show resemblance to primary demyelination due to defective or improper myelin formation as in Canavan's disease and adreno‐myelopathy in man and associated experimental mouse models (Baumann & Pham‐Dinii, 2001); polyunsaturated fatty acids, including essential fatty acids (EFA), are highly vulnerable to irradiation due to an increase in lipid peroxide formation (Hammer & Wills, 1979), reflected by higher thiobarbituric acid reactive substances (TBARS) and nuclear magnetic resonance (NMR) values after irradiation (De Oliveira Silva, Mársico, Oliveira de Jesus, Guimarães, & Sloboda Cortez, 2011) mostly affecting polyunsaturated fatty acids resulting in more saturated fatty acids (Javan & Motallebi, 2015), and increased formation of trans fatty acids (Yilmaz & Geçgel, 2007); nervous tissue concentrates omega‐3 polyunsaturated fatty acids in myelin (Lenox, 2015); lipid composition and fatty acid profiles of myelin of rat brain vary in response to the consumption of different fats (Bourre et al., 1984; Srinivasarao, Vajreswari, Rupalatha, & Narayanareddy, 1997); and induction of EFA deficiency in rats resulted in both a decrease of EFA in myelin but also vacuolation/myelin splitting in the optic nerve (Trapp, 1977), we hypothesized that abnormalities in fatty acid metabolism in myelin might play an essential role in the pathogenesis of this acquired form of leukoencephalomyelopathy in cats. Therefore, we investigated in addition the fatty acid profile of the white matter of the spinal cord of affected cats and control animals, and of irradiated and non‐irradiated food.

## 2 MATERIALS AND METHODS

### 2.1 Animals

In July–August 1989, 12 out of 33 cats in a breeding colony developed progressive paresis/ paralysis and ataxia. The colony was maintained unvaccinated in a maximum barrier production unit and received irradiated dry cat food. Cats were between 2 months and 2 years of age, and affected animals were not genetically related and included both males and females. Cats otherwise had normal appetite, water consumption, reproduction and behaviour. From July to October 1993, a second outbreak affecting six out of 21 cats occurred in a group of SPF cats from the same breeding facility as well as in six animals transferred to a research laboratory. Also, these cats were maintained unvaccinated in a maximum barrier production unit and received irradiated dry cat food. In June–July 1997, a third outbreak was observed in the same breeding colony and affected 40 out of 60 females and males. A fourth outbreak with similar symptoms was observed in September–October 2000, in a different breeding colony consisting of 20 females and three males, and finally affecting 80% of the animals, as well as in January 2001 in a group of eight cats transferred from this colony to a research laboratory for a behavioural study. Also, these animals were held under SPF conditions and received irradiated dry cat food. In all these outbreaks severely affected animals died or were euthanized. Mildly affected animals showed gradual improvement/disappearance of symptoms within 4–10 months after receiving a different batch of food. The three control cats, which were used for fatty acid determinations of the spinal cord white matter, were a private owned queen of 10 months old with acute cardiac decompensation after ovariohysterectomy and two healthy adult queens used as control cats in an experimental feline infectious peritonitis model study.

### 2.2 Post‐mortem examination

Thirteen severely affected cats from the various outbreaks between 1989 and 1997, as well as six out of eight cats from the outbreak in January 2001, the latter all euthanatized after clinical signs developed within the group, were available for post‐mortem examination. Complete post‐mortem examination was performed by board‐certified veterinary pathologists. Samples for histological examination were taken from tonsil, mandibular or pre‐scapular lymph node, (para)thyroid gland, thymus, heart, lung, liver, spleen, kidney, adrenal gland, stomach (fundus), pancreas, duodenum, colon, ovary and uterus respectively testicle and skeletal muscle (M. semitendinosus) and in the cats from the 2001 outbreak also from subcutaneous (inguinal) and perirenal fat tissue. Samples were fixed in 10% neutral, phosphate‐buffered, formalin. For neuropathological examination, brain, spinal cord including spinal ganglia, right brachial and sciatic nerves, coeliac ganglion, trigeminal ganglion and pituitary gland were fixed by immersion in 10% neutral, phosphate‐buffered, formalin. After routine dehydration and embedding in paraffin, 4 µm sections were made and stained with haematoxylin and eosin (HE). Specific staining methods used in selected slides from the nervous system were toluidine blue and immune‐histochemical stains for glial fibrillary acidic protein (GFAP), ionized calcium binding adaptor molecule 1 (IBA‐1; a microglial and histiocyte marker) and alpha‐smooth muscle actin (αSMA) using standard procedures.

### 2.3 Fatty acid determinations in spinal white matter and food

Spinal white matter was collected from three affected cats from the 1997 outbreak and six affected cats from the outbreak in 2001 as well as from the three control cats. Spinal cord material was frozen in liquid nitrogen and stored at −70°C until use. Immediately prior to analysis the material was thawed and ground. Also, a sample of the irradiated food associated with the 2000 outbreak (SSNIFF, 2.5Mrad = 25 kGy) and of a standard dry cat food from the same manufacturer were obtained and ground prior to analysis.

Cholesteryl esters (CE) were isolated by a modified “Bligh & Dyer” extraction according to Retra et al. (2008), followed by a solid‐phase extraction method according to Hamilton and Comai (1988). Thereafter the CEs were saponified according to the modified method described by Kates (1986), where petroleum ether was replaced by hexane. Polyunsaturated fatty acid analysis was performed by high‐pressure liquid chromatography/mass spectrometry (HPLC/MS) according to Retra et al. (2008) in which the Synergi 4 µm MAX‐RP 18A column was replaced by a Kinetex 2.6 µm C18 100A column (150 × 3 mm; Phenomenex, CA, USA). Internal standards were used for comparison.

## 3 RESULTS

### 3.1 Pathomorphological findings

All animals were in good bodily condition, and no gross abnormalities were seen.

**Figure 1:**
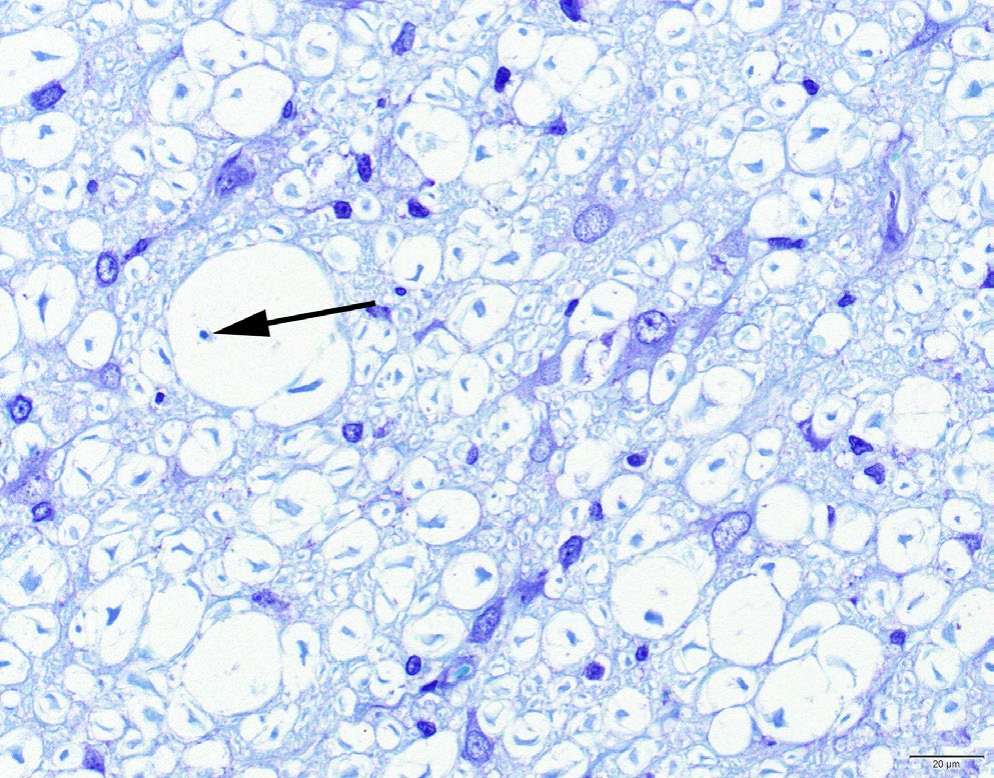
Cat. Leukoencephalomyelopathy. Spinal cord white matter with vacuolation and areas of approximation of myelin sheaths and associated astrocytosis, microgliosis and capillary hyperplasia. The vacuoles often contain intact axons but sometimes also loss of the axon and presence of a myelophage (arrow) is observed. Toluidine blue [Colour figure can be viewed at http://wileyonlinelibrary.com]

**Figure 2:**
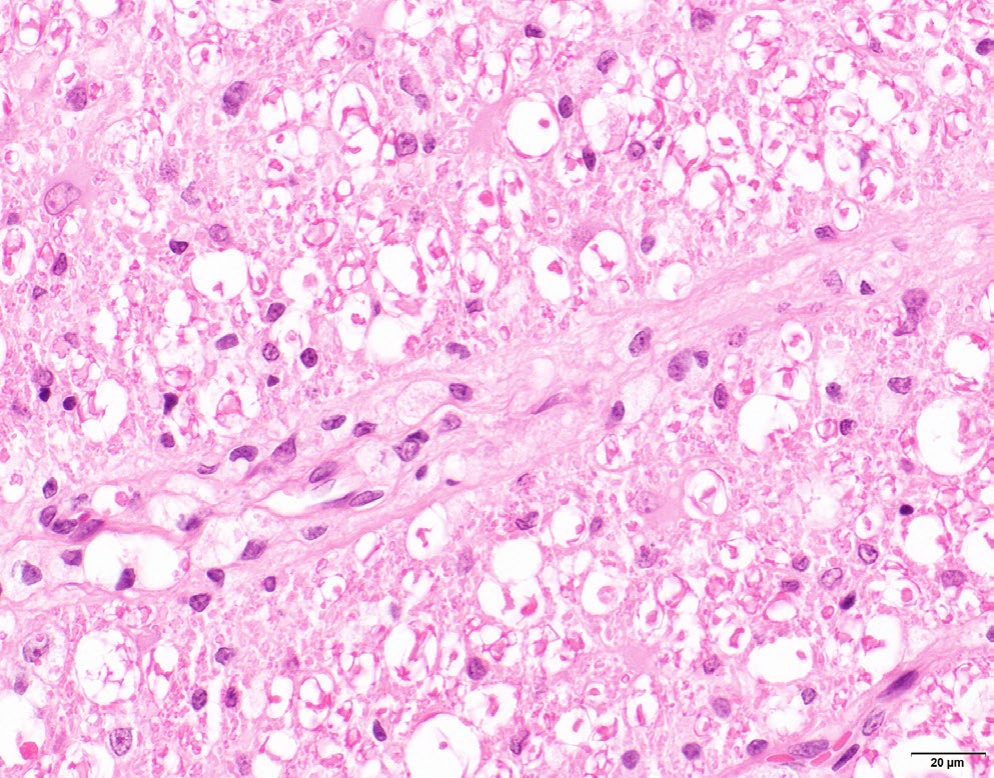
Cat. Leukoencephalomyelopathy Spinal cord white matter. Perivascular cuffing with large foamy lipid‐containing cells. HE [Colour figure can be viewed at http://wileyonlinelibrary.com]

Histologically, lesions were consistently present in the central nervous system and comparable in distribution and severity in all severely affected cats. In both the brain and the spinal cord, lesions were restricted to the white matter, and consisted of bilateral symmetric vacuolation, loss of myelin and variously associated astrocytosis, microgliosis and capillary hyperplasia. The vacuoles often contained intact and sometimes swollen axons but also loss of axons associated with cellular debris and/or myelophages was observed (Figure 1). Besides vacuolation, areas with approximation of axons possibly due to small‐sized myelin sheaths were regularly observed in the white matter of the spinal cord (Figure 1). Astrocytes were enlarged with pale‐pink cytoplasm, large nuclei and prominent nucleoli. In severely affected areas, perivascular cuffs as well as parenchymal solitary large foamy lipid‐containing cells were observed (Figure 2). Rarely, a small perivascular lymphocytic infiltrate was seen in only two cats. In the cats from the most recent outbreak in January 2001, the spinal cord lesions were severe in all segments of the spinal cord in four cats, whereas in the fifth cat, moderate lesions were present in the thoracic area and slight lesions in the lumbal area, and in the sixth cat, almost no abnormalities in the thoracic and lumbal areas were seen.

The lesions in the white matter had a particular and consistent distribution. In the cerebrum, marked lesions were seen in the white matter of the corona radiata, the outer cortex, the capsula interna and in the optic tract. The corpus callosum and the thalamic white matter showed no or only minimal lesions. In the mesencephalon, lesions were almost restricted to the corticospinal tracts and the rostral colliculi. The white matter of the cerebellar cortex showed no or minimal abnormalities, moderate lesions were seen in the white matter surrounding the cerebellar nuclei. In the pons and medulla oblongata, moderate to marked lesions existed in the vestibular, spinocerebellar and pyramidal tracts, the white matter of the formatio reticularis showed no abnormalities. In the spinal cord extensive lesions were found over the full length of the spinal cord. Dorsal, lateral and ventral funiculi were almost equally affected, although astrogliosis, microgliosis and perivascular cuffing with foamy lipid‐containing cells were most prominent in the lateral and ventral funiculi. Despite the extent of the lesions in the white matter of the spinal cord, the fasciculi proprii seemed spared. The spinal ganglia and the afferent and efferent nerves of the spinal cord, the peripheral nerves (brachial and ischiadic, intramuscular and intrinsic nerves) as well as the coeliac and trigeminal ganglia showed no abnormalities.

**Figure 3:**
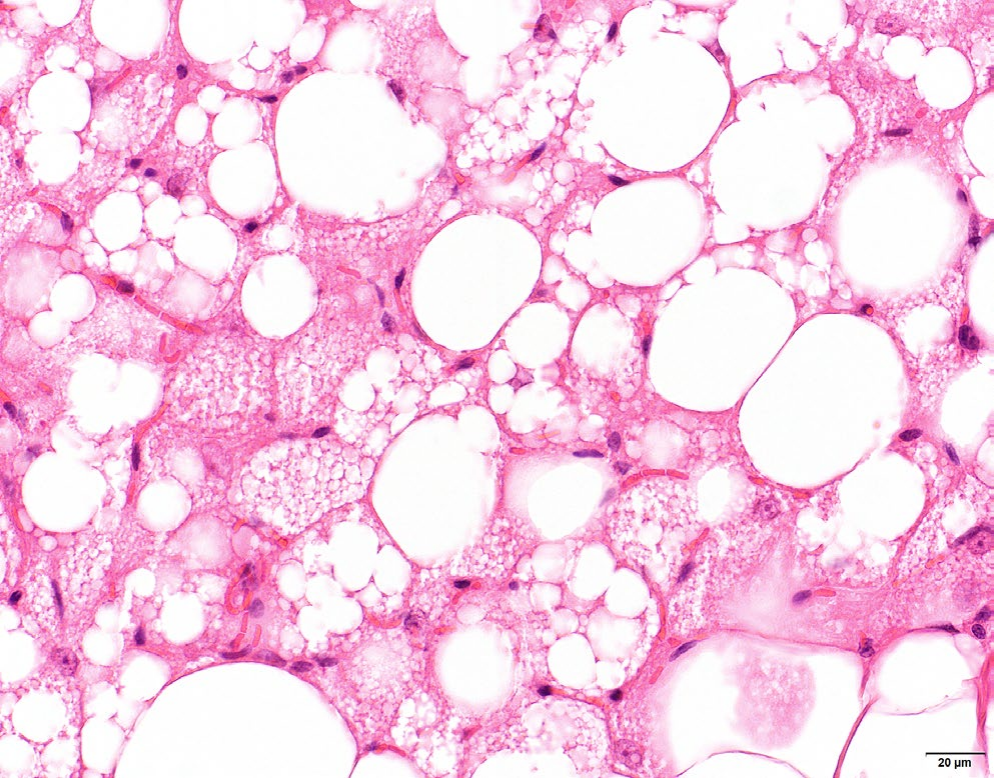
Cat. Inguinal subcutaneous adipose tissue. Lipolysis characterized by multi‐vacuolar adipocytes in between normal uni‐vacuolar adipocytes. HE [Colour figure can be viewed at http://wileyonlinelibrary.com]

In the adipose tissue, only sampled in the animals from the January 2001 cases, a peculiar additional finding was the presence of single or groups of multi‐vacuolar adipocytes (Figure 3) at various sites in the body (inguinal subcutaneous, perirenal, mediastinal and mesenteric adipose tissue) in all cats severely affected, and no respectively minor changes in the cats with lesser spinal cord lesions. No significant abnormalities were observed in the other organs.

**Figure 4:**
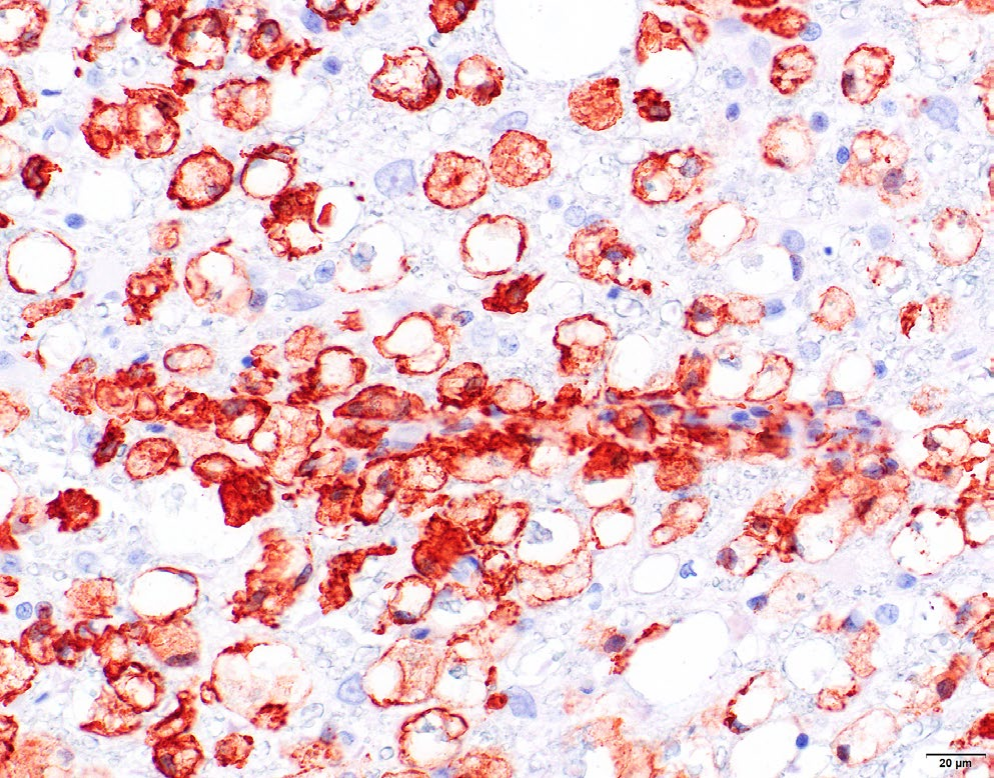
Cat. Leukoencephalomyelopathy. Spinal cord white matter. Marked proliferation of microglia with marked positive staining of perivascular cuffs and parenchymal solitary lipid‐containing microglial cells. Immunohistochemistry for IBA‐1 [Colour figure can be viewed at http://wileyonlinelibrary.com]

**Figure 5:**
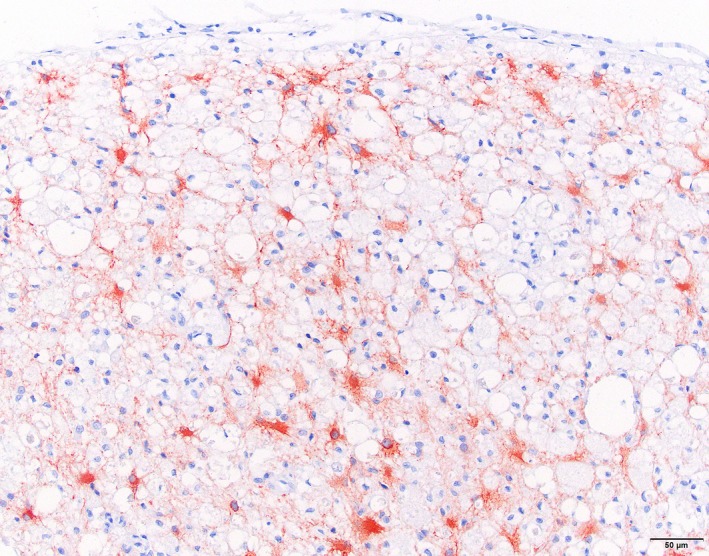
Cat. Leukoencephalomyelopathy. Spinal cord white matter. Marked staining in the affected white matter with swollen astrocytes and extensive proliferation of astrocytic processes. Immunohistochemistry for GFAP [Colour figure can be viewed at http://wileyonlinelibrary.com]

Immunohistochemistry for IBA‐1 showed marked microglial proliferation in the affected areas, variable staining of myelophages in vacuolated myelin sheets and positive staining of the perivascular cuffs of lipid‐containing cells as well as solitary parenchymal lipid‐containing cells (Figure 4). GFAP showed marked staining in the affected white matter with swollen astrocytes and extensive proliferation of astrocytic processes (Figure 5). αSMA showed staining of arteriolar smooth muscle as well as in smaller vessels of individual pericytes/smooth muscle cells comparable with the normal spinal cord.

### 3.2 Fatty acid composition spinal white matter and irradiated and non‐irradiated food

The fatty acid composition of the spinal white matter of nine affected cats as well as of three control cats is given in Table 1. Affected cats had higher levels of docosadienoic acid (C22:2n‐6), docosatetraenoic acid (C22:4n‐6) and unknown fatty acids, and lower levels of myristic acid (C14:0), palmitoleic acid (C16:1n‐9), linoleic acid (C18:2n‐6), and even absence of alpha‐linolenic acid (C18:3n‐3) in their spinal white matter. The fatty acid composition of the irradiated diet before and after irradiation is given in Table 2. Total dietary fat content was 10% (100.0 g per kg) for both the irradiated and the non‐irradiated food. Irradiation of the food resulted in an altered fatty acid profile, as demonstrated by higher levels of lauric acid (C12:0), myristic acid (C14:0), palmitic acid (C16:0), stearic acid (C18:0), arachidonic acid (C20:4n‐6), eicosapentaenoic acid (C20:5n‐3), docosahexaenoic acid (C22:6n‐3) and lower levels of pentadecanoic acid (C15:0), palmitoleic acid (C16:1n‐9), oleic acid (C18:1n‐9), linoleic acid (C18:2n‐6), alpha‐linolenic acid (C18:3n‐6), arachidic acid (C20:0), paullinic acid (C20:1n‐7), eicosadienoic acid (C20:2n‐6), eicosatrienoic acid (C20:3n‐6), arachidonic acid (C20:4n‐6), eicosatrienoic acid (C20:3n‐3), erucic acid (C22:1n‐9), adrenic acid (C22:4n‐6) and lignoceric acid (C24:0) as well as a high total omega‐6:omega‐3 ratio in the irradiated food of 7:1.

**Table 1:** Fatty acid composition of the spinal white matter expressed as percentage of total fatty acids

Fatty acid	Average affected cats	*SD*	Average control cats	*SD*
12:0	0.00	0.00	0.00	0.00
14:0	0.59	0.05	0.78	0.15
14:1	0.00	0.00	0.00	0.00
15:0	0.37	0.06	0.13	0.02
16:0	19.34	1.04	19.32	0.21
16:1	0.34	0.26	0.82	0.13
17:0	0.13	0.21	0.27	0.03
17:1	1.01	0.04		
18:0	17.71	0.95	17.04	0.41
18:1 n‐9	18.62	2.45	18.95	1.36
18:1 n‐7	4.08	0.18	3.53	0.12
18:2 n‐6 trans	1.52	0.17	4.57	1.03
18:3 n‐6	0.01	0.03	0.00	0.00
18:3 n‐3	0.00	0.00	0.25	0.07
20:0	0.05	0.08	0.13	0.01
20:1	1.32	0.36	0.92	0.21
20:2 n‐6	0.10	0.15	0.21	0.09
20:3 n‐6	0.79	0.16	0.53	0.14
20:4 n‐6	7.62	1.39	6.25	1.49
20:3 n‐3	0.00	0.00	0.00	0.00
20:5 n‐3	0.00	0.00	0.00	0.00
22:1 n‐9	0.06	0.09	0.13	0.03
22:2	0.05	0.08	0.02	0.04
22:3	0.65	0.14		
22:4 n‐6	2.96	0.51	2.16	0.51
22:5	0.29	0.32	0.76	0.43
24:0	0.10	0.08	0.00	0.00
24:1	0.00	0.00		
22:6 n‐3	11.61	0.99	13.22	1.86
Unknown	12.05	1.87	9.98	0.65

**Table 2:** Fatty acid composition of the irradiated (SSNIFF 2,5Mrad = 25 kGy) and non‐irradiated food (from same manufacturer) expressed as percentage of total fatty acids

Fatty acid	Non‐irradiated	Irradiated
12:0	0.20	0.41
14:0	1.00	3.27
14:1 n‐9	NA	0.29
15:0	0.20	0.04
16:0	19.70	22.18
16:1 n‐9	3.80	2.44
17:0	0.40	0.4
18:0	5.20	9.43
18:1 n‐9	34.80	32.06
18:1 n‐7	NA	2.42
18:2 n‐6	26.30	21.85
18:3 n‐3	2.90	1.38
20:0	0.20	0.09
20:1 n‐7	1.00	0.6
20:2 n‐6	0.60	0
20:3 n‐6	0.20	0
20:4 n‐6	0.10	0.25
20:3 n‐3	0.40	0
20:5 n‐3	0.60	0.85
22:1 n‐9	0.80	0
22:4 n‐6	0.20	0
22:5 n‐6	NA	0.44
24:0	0.10	0
22:6 n‐3	0.80	1.09
Unknown	NA	0.89
Total fat content	100.0 g per kg	100.0 g per kg

## 4 DISCUSSION

The irradiated food‐induced leukoencephalomyelopathy in cats as demonstrated in our study has some special characteristics and thereby differs from many other forms of demyelination. It is an acquired disease that affects only cats and develops after feeding the irradiated food for four to six months. The disease, at least in the early clinical stage, is morphologically characterized by myelin vacuolation and demyelination with some myelophages but in general in the presence of intact axons, as well as by remyelinization with thin myelin sheaths around intact axons. When the cats at this stage are returned to a normal food, the animals show complete clinical recovery after 6–8 weeks although after 12 and 24 months these recovered cats still show thin myelin sheaths characteristic for remyelinated axons (Duncan, Marik, Broman, & Heidari, 2017). As already suggested by Duncan et al. (2009), the disease therefore may be regarded as a primary disease of the myelin sheath/oligodendrocyte. Defective myelination may disrupt axo‐glial cell interactions, axolemmal organization and electrical conduction and connectivity and hence in chronic cases may cause axonal damage and loss affecting the specific biophysical properties of myelin as an electrical insulator (Piaton, Gould, & Lubetzki, 2010). The observed reactive astrocytosis and microglial and endothelial proliferation most likely are secondary phenomena.

The myelin sheath is an extended highly specialized plasma membrane synthesized in the central nervous system by oligodendrocytes. One of the prominent biochemical characteristics that distinguishe myelin from other membranes is its high lipid‐to‐protein ratio. Lipids account for at least 70% of the dry weight of myelin membranes. Much of the structure and function of myelin is dependent on its lipid content. The lipid characteristics of myelin together with the presence of myelin‐specific proteins are likely required for myelin wrapping and compaction to confer to the specific biophysical properties of myelin as an electrical insulator (Chrast, Saher, Nave, & Verheyen, 2011). Mature compacted myelin in rats is markedly enriched in saturated and monosaturated fatty acids, especially lignoceric acid (C24:0) and nervonic acid (C24:1n‐9), whereby the maturity and compaction of myelin is assessed by the ratio of lignoceric acid (C24:0) + nervonic acid (C24:1n‐9) to stearic acid (C18:0) + oleic acid (C18:1n‐9). In fact, the accumulation of these marker very long‐chain fatty acids (VLCFA) determines the process of myelin maturation in rats (Chrast et al., 2011; Srinivasarao et al., 1997).

A remarkable finding with respect to the fatty acid composition of the white matter of the spinal cord in affected animals compared to normal cats were the low levels of linoleic acid (C18:2n‐6) and even absence of alpha‐linolenic acid (C18:3n‐3) and the high levels of docosadienoic acid (C22:2n‐6), docosatetraenoic acid (C22:4n‐6) and unknown fatty acids in the affected cats. As myelin composition is directly influenced by the composition of dietary fats (Bourre et al., 1984; Srinivasarao et al., 1997), this might indicate a deficient supply of linoleic acid (C18:2n‐6) and particularly alpha‐linolenic acid (C18:3n‐3) from the irradiated food in our cats. Another remarkable finding with respect to the fatty acid composition of the white matter of the spinal cord in both affected and normal cats in our study was the absence of lignoceric acid (C24:0) as well as the absence of nervonic acid (C24:1), characteristic markers of mature compacted myelin in other animal species and man. To maintain the electrophysiological properties of myelin in cats, a replacement of these saturated and monosaturated VLCFAs by very long‐chain polyunsaturated fatty acids (VLC‐PUFAs) is necessary which, compared to long‐chain polyunsaturated fatty acids (LC‐PUFAs), have the potential to provide distinct roles in membrane structure and fluidity behaving like unusual saturated fatty acids. VLC‐PUFAs may allow for distinct membrane fluidity and packing density, creating a unique lipid bilayer that is potentially beneficial for the compaction of myelin (Murray, Walchuk, & Suh, 2018). However, the VLCFAs lignoceric acid (C24:0) and nervonic acid (C24:1n‐9) are synthesized locally by elongation from the saturated and monosaturated long‐chain fatty acids stearic acid (C18:0) and oleic acid (C18:1n‐9), many studies have substantiated that VLC‐PUFAs are synthesized locally through a series of elongation and desaturation steps from available shorter‐chain polyunsaturated fatty acids (Murray et al., 2018) whereby elongation preferentially occurs from the n‐3 fatty acids particularly eicosapentaenoic acid (20:5n‐3; Yu et al., 2012). The absence of alpha‐linolenic acid in the white matter of the affected cats may therefore also reflect an increased need for alpha‐linolenic acid as a substrate for longer chain omega‐3 fatty acids to compose myelin sheaths and thus indicate an abnormal sensitivity for a deficient supply in the food of polyunsaturated fatty acids particularly omega‐3 polyunsaturated fatty acids in cats.

Total fat content of the food was 100.0 g per kg dry matter basis, which is close to the minimum recommended amount by FEDIAF nutritional guidelines publication December 2018 (i.e., 90.0 g per kg dry matter basis). The alpha‐linolenic acid (C18:3n‐3) content of the irradiated food is low compared to the amount of linoleic acid. Although alpha‐linolenic acid is not regarded an essential nutrient for adult cats, it is regarded as such for growing kittens (FEDIAF nutritional guidelines publication December 2018). The total omega‐6:omega‐3 ratio in the irradiated food is 7:1, whereas nutritionists typically recommend ratios between 2:1 and 1:1 for cat food. Such increased total omega‐6:omega‐3 ratio in the irradiated food might contribute to a relative deficiency of alpha‐linolenic acid (C18:3n‐3), as there is competition for metabolic enzymes with omega‐6 fatty acids such as linoleic acid (C18:2n‐6). Different omega‐6:omega‐3 ratios are reported for different indications (Simopoulos, 2002), and not only omega‐6:omega‐3 ratio is important, as there needs to be a certain amount of eicosapentaenoic acid (C20:5n‐3) and docosahexaenoic acid (C22:6n‐3) present in the diet to achieve effective plasma levels (Hall, Picton, Skinner, Jewell, & Wander, 2006).

Oligodendrocytes in the adult are thought to primarily rely on uptake to maintain their fatty acids pool (Dimas, 2017). Oligodendrocytes with a shortage in lipid synthesis appear to be supported by horizontal lipid flux from astrocytes (Camargo et al., 2017). Failure of astrocytes to provide this support results in increased uptake of circulating usually dietary lipids by myelinating oligodendrocytes (Camargo et al., 2017). In case of a deficient dietary supply of specific lipid components like n‐3 polyunsaturated fatty acids, these may be released by lipolysis from perineural adipocytes for peripheral nerve lipid supplementation (Montani et al., 2018) or, as probably is the case in the extensive and severe leukoencephalomyelopathy in cats on an irradiated diet, from the fat deposits in the body. The extensive presence of multivesicular adipocytes, characteristic for lipolysis, in the fat tissues in the cats in the most recent outbreak supports our view of a dietary deficiency of lipid components in the irradiated food as cause for the development of the leukoencephalomyelopathy in cats fed on a long‐term diet of irradiated dry cat food. The perivascular cuffs and parenchymal solitary lipid‐containing microglial cells in the affected white matter in cats with leukoencephalomyelopathy probably reflect an influx of lipids/fatty acids from the circulation to provide a substrate for astrocytes and oligodendrocytes.

Increased peroxide levels in the spinal cord of affected cats as demonstrated by Caulfield et al. (2008) in our opinion are not a cause but a secondary phenomenon as abnormal fatty acid composition in murine oligodendrocytes has been described to induce oxidative stress and overproduction of reactive oxygen species (Baarine et al., 2012).

However, the myelin sheath has been regarded for a long time as an inert insulating structure, it has now become clear that myelin is metabolically active. Even if the turnover of lipids in myelin is slow with a half‐life of several weeks to months, there is a constant exchange of molecules (Schmitt, Castelvetri, & Simons, 2015; Simons & Nave, 2015). This may explain the long, 4–6 months, period for the development of clinical disease in cats exposed to the irradiated dry cat food.

Apart from domestic cats also other wild felidae may be more sensitive to these alterations in dietary fatty acid pattern as a comparable form of leukoencephalomyelopathy has been described in captive cheetahs and other large felids (Brower et al., 2014; Palmer et al., 2001). Unfortunately, in these cases the diet composition was unknown. However, nutritional deficiencies are common in wild animals that are kept in captivity (Crissey, 2005), so a deficient or unbalanced fatty acid pattern of the diet also might be at the origin of the leukoencephalomyelopathy in these wild felids.
